# Editorial: Unraveling diarrheic virus-host interactions: mechanisms and implications

**DOI:** 10.3389/fcimb.2025.1684757

**Published:** 2025-08-29

**Authors:** Ahmed H Ghonaim, Yanrong Zhou, Gaopeng Hou, Yinxing Zhu, Wentao Li

**Affiliations:** ^1^ National Key Laboratory of Agricultural Microbiology, Hubei Hongshan Laboratory, College of Veterinary Medicine, Huazhong Agricultural University, Wuhan, China; ^2^ Department of Molecular Microbiology, Washington University School of Medicine, St. Louis, MO, United States

**Keywords:** viral gastroenteritis, virus-host interaction, gut microbiota, antiviral compounds, diagnostic assays

Understanding the complex interplay between diarrheic viruses and their hosts is central to combating the global burden of viral gastroenteritis in both humans and animals. The Research Topic *“Unraveling Diarrheic Virus–Host Interactions: Mechanisms and Implications”* presents seven diverse articles that span molecular virology, host immune modulation, diagnostic innovation, gut microbiota dysbiosis, and outbreak investigation. Together, these studies provide fresh insights into virus–host dynamics and point toward translational strategies for diagnosis, prevention, and treatment.

## Viruses and the gut ecosystem: from microbiota to metabolomics

Lv et al. reviewed the diverse factors shaping piglet gut microbiota, such as host genetics, maternal influences, feeding environment, diet, and the pathogenic challenge of porcine epidemic diarrhea virus (PEDV). The authors’ analysis highlighted how PEDV disrupts the intestinal barrier and microbial balance. They also discussed the potential of Chinese herbal medicine, particularly *Qiwen Huangbai San*, to restore mucosal immunity and promote microbial homeostasis.

To investigate the role of the gut microbiota in human viral gastroenteritis, Wang et al. conducted a metagenomic study of children infected with norovirus. They found persistent dysbiosis, enrichment of *Bacteroides uniformis* and *Veillonella*, and altered carbohydrate and lipid metabolic pathways that correlated with disease severity. These findings offer candidate biomarkers for diagnosis and therapy.

## Host-targeted antivirals and mechanistic insights

The antiviral potential of *Saxifraga stolonifera* was explored by Lu et al., who demonstrated that this plant disrupts the interaction between the PEDV nucleocapsid protein and host p53. The researchers identified quercetin and other bioactive components as key effectors and, through network pharmacology and molecular docking, linked these compounds to modulation of p53-related signaling pathways, highlighting a host-targeted antiviral approach.

Wakeford et al. reported that mutation of the K48 ubiquitin linkage site in host cells markedly reduced the replication of murine norovirus. This was achieved by creating a non-permissive, pro-inflammatory environment, which revealed the importance of ubiquitination dynamics in viral propagation.

## Surveillance, epidemiology, and diagnostic innovation

Long-term epidemiological monitoring of norovirus in Shenzhen was carried out by Wang et al., who analyzed seven years of surveillance data. Their study uncovered genotype shifts, recombination breakpoints, and mutations associated with viral evolution, with infections concentrated among children under three years of age and peaking during the winter in more developed districts.

In an outbreak investigation, Li et al. identified airborne transmission from vomitus exposure as the main route of spread for sapovirus in a Shenzhen school. The authors showed that prompt decontamination and adherence to standard vomit cleanup protocols significantly reduced the number of cases.

Wang et al. developed a multiplexed TaqMan MGB qPCR assay for the simultaneous detection of four major feline viruses. The assay demonstrated high sensitivity, specificity, and throughput, enabling rapid diagnostics in multi-pathogen infection scenarios, which are becoming increasingly common in clinical veterinary practice.

## Perspectives and future directions

Taken together, these articles underscore the complexity and translational potential of research into diarrheic virus–host interactions. Several unifying themes emerge:

Microbiota–immune crosstalk is both a driver and a consequence of viral infections.

Host-directed antivirals, whether herbal-derived or targeting ubiquitin pathways, show promise in reducing resistance development.

Integrated surveillance and innovative diagnostics remain central to early detection and containment, especially in high-density or zoonotic contexts.

Looking ahead, integrating systems biology, multi-omics, structural virology, and interactomics will be crucial for identifying universal host targets and novel therapeutics. Bridging laboratory discoveries with field-ready applications will accelerate progress in controlling viral gastroenteritis across species.

